# Statistical average strain energy density fatigue estimation of strut-based metamaterials via synthetic as-built CAD digital twins

**DOI:** 10.1038/s44455-026-00030-z

**Published:** 2026-06-02

**Authors:** Simone Murchio, Raffaele De Biasi, Marcello Laurenti, Nicolò Bonato, Simone Carmignato, Matteo Benedetti, Filippo Berto

**Affiliations:** 1 Department of Chemical Engineering Materials Environment, La Sapienza University of Rome, Roma, Italy; 2 Department of Industrial Engineering, University of Trento, Trento, Italy; 3 Department of Management and Engineering, University of Padova, Vicenza, Italy

**Keywords:** Engineering, Materials science

## Abstract

The limited and scattered fatigue performances and their difficult predictability remain critical barriers for the widespread adoption of Laser-based Powder Bed Fusion (L-PBF) metamaterials in engineering applications, as fatigue damage initiation is highly sensitive to manufacturing-induced geometric imperfections. While X-ray computed tomography (CT) provides high-fidelity as-built reconstructions fundamental for metamaterials’ structural health monitoring, its cost and complexity hinder routine integration into fatigue assessment workflows at the design stage. In this work, we propose a computationally efficient framework for the development of synthetic as-built CAD models, serving as digital twins for fatigue life and failure location prediction. The proposed model is herein reported for L-PBF Ti-6Al-4V struts, the elemental building blocks of metamaterial architectures, manufactured at different building orientations. Leveraging stereomicroscopy input images, a modular reconstruction pipeline capturing orientation-dependent surface morphology and partially fused particles allows the generation of as-built CAD models that retain the geometric variability governing fatigue behaviour, without reliance on volumetric imaging. Synthetic models are coupled with finite element analyses and a statistical strain energy density criterion to identify failure-critical locations. Validation against CT-derived counterparts demonstrates close morphological agreement and, since the design stage, the ability to estimate fatigue life and predict experimental failure locations within established scatter bands.

## Introduction

Architected mechanical metamaterials enabled by metal additive manufacturing (AM) offer unprecedented opportunities to tailor stiffness, strength, and failure behaviour through geometry rather than composition^1,2^. Among these systems, strut-based lattice architectures fabricated by laser powder bed fusion (L-PBF) have emerged as prime candidates for lightweight, fatigue-critical applications due to their high structural efficiency and geometric flexibility^2^. However, the same geometric complexity and process-induced imperfections that underpin their performance also render fatigue behaviour highly sensitive to local features, posing significant challenges to the development of reliable, design-oriented predictive models^3,4^.

This difficulty arises from the intrinsic coupling between lattice geometry, manufacturing-induced defects, and the resulting local stress-strain fields^5^. In L-PBF metamaterials, thin struts, high surface-to-volume ratios, orientation-dependent surface roughness, and process-induced porosity lead to pronounced deviations of the as-built geometry from nominal computer-aided design (CAD) representations. Experimental and numerical studies have consistently demonstrated that strut waviness, cross-sectional variability, surface-adhered particles, and internal pores act as dominant stress concentrators, governing fatigue crack initiation and early propagation in lattice architectures^6,7^. Consequently, fatigue life and failure location are dictated by highly localized, defect-sensitive mechanisms rather than by nominal or homogenized stresses at the lattice scale, limiting the applicability of conventional fatigue design approaches originally developed for smooth or notched bulk components^8^.

To capture these effects, high-resolution X-ray computed tomography (CT) has become the reference technique for reconstructing the as-built geometry and internal defect population of AM lattices. CT-based numerical models have shown substantially improved agreement with experimental mechanical and fatigue behaviour compared to simulations based on ideal CAD geometries, highlighting the decisive role of as-manufactured features^6,9^. However, CT-centred workflows remain costly, time-intensive, and thus challenging to integrate into routine design loops, as they demand extensive computational resources for geometry reconstruction and high-fidelity meshing^10^. These limitations are particularly restrictive for mechanical metamaterials, where performance-driven design requires evaluating multiple architectures, build orientations, and defect realizations. Although recent efforts have explored digital twins and statistically equivalent geometrical representations to mitigate the computational burden associated with fully resolved simulations, many of these approaches still rely on high-resolution CT models as their primary source of geometric input, thereby shifting, rather than eliminating, the characterization bottleneck^7,11^. In particular, stochastic and statistically informed reconstructions of as-built strut geometries, such as those proposed by *Lozanovski* et al.^12^, are derived from statistical descriptors extracted from CT scans. Similarly, digital-twin frameworks developed in^4,13^ rely on extensive CT-based reconstruction and segmentation to quantify strut- and node-level variability and to enable non-destructive certification of lattice structures. While these CT-driven strategies have demonstrated excellent predictive capability for stiffness, buckling, and functional response, their dependence on detailed tomographic data remains a limiting factor for routine, fatigue-critical assessments and large-scale design exploration. There is therefore a clear need for predictive frameworks that retain the essential physics of fatigue damage in AM lattices while remaining computationally and experimentally efficient.

Complementary strategies to bypass fully resolved CT-based reconstructions have therefore focused on stochastic and statistically equivalent representations of as-built strut geometries^14,15^. Monte Carlo-based frameworks enable the generation of synthetic digital realizations of AM struts that can be embedded into computationally efficient beam-based lattice models, allowing large-scale simulations while accounting for manufacturing-induced geometric variability^12^. Related efforts have also explored virtual manufacturing approaches, in which process-induced geometric defects are predicted using physics-based models and subsequently incorporated into numerical analyses^16^. While these contributions clearly demonstrate the potential of synthetic as-built models, their calibration and validation typically remain still tied to tomographic measurements, limiting their applicability for fatigue-critical assessments and extensive design exploration.

Building on this perspective, a promising strategy to address fatigue prediction is to adopt a bottom-up approach focused on the sub-unit strut elements that fundamentally govern the mechanical and fatigue response of lattice architectures. Fatigue in AM lattices is a highly localized phenomenon, with crack initiation and early propagation typically occurring within individual struts or at strut junctions, well before any global lattice-scale failure develops. Experimental investigations on miniaturized L-PBF Ti-6Al-4V struts have shown that fatigue life is strongly controlled by local surface morphology, cross-sectional variability, and defect population, with pronounced dependencies on building orientation and loading conditions^17–19^. These findings support the notion that modelling the fatigue behaviour of individual struts provides a physically grounded and scalable route to predicting the durability of lattice-based metamaterials. This conclusion is further supported by studies comparing struts with and without junctions, which have shown that nodes act as additional stress concentrators, often reducing fatigue strength relative to plain struts while modulating sensitivity to building orientation and surface irregularities^17,20^. At the same time, these works confirm that dominant fatigue mechanisms remain local and geometry-driven, reinforcing the suitability of strut-level analyses as a foundation for lattice-scale fatigue assessment. Within this context, energy-based fatigue parameters are especially attractive, as they naturally account for multiaxial stress states, mean stress effects, and notch sensitivity, features that are ubiquitous in AM thin struts with irregular as-built surfaces. Recent fatigue studies on miniaturized L-PBF struts subjected to different stress ratios have highlighted the strong influence of mean stress, surface condition, and defect location on fatigue strength, while showing that classical stress-based criteria alone are insufficient to rationalize the observed trends^19^.

Among energy-based approaches, the Average Strain Energy Density (ASED) criterion has proven particularly effective for predicting fatigue strength and life in notched metallic components and AM materials^21^. The ASED framework postulates that fatigue damage is governed by the elastic strain energy averaged over a finite control volume surrounding critical stress raisers, rather than by local peak stresses alone. This formulation provides a robust link between local stress-strain fields and fatigue behaviour, while significantly reducing mesh dependency and numerical sensitivity to stress singularities, which are especially pronounced in AM components with irregular surfaces and sharp geometric features. ASED has been successfully applied to a wide range of fatigue problems in AM metals and, more recently, to cellular and lattice materials when combined with detailed CT-based reconstructions and probabilistic frameworks accounting for defect statistics and extreme value effects^22^. Mean stress effects can also be incorporated directly within modified strain energy density formulations, further enhancing their applicability to complex loading conditions^23^. Despite the demonstrated accuracy of these approaches, their reliance on high-fidelity as-built geometrical representations, often obtained via full CT reconstruction or image-based numerical methods, limits their applicability in routine design workflows due to computational cost^24^. In parallel, recent studies have shown that ASED can be effectively integrated within reduced-order and compensated beam frameworks, enabling design-oriented analyses and specimen optimization while retaining physical fidelity^25^. Nevertheless, the systematic application of ASED to fatigue prediction in architected metamaterials using simplified, synthetic as-built geometrical models, rather than CT-derived reconstructions, remains largely unexplored.

In this work, we propose a cost-effective and mechanics-based framework for fatigue life and failure location prediction in L-PBF strut-based metamaterials. The approach combines synthetic as-built CAD models of miniaturized struts, generated from stereomicroscopy imaging and surface profile reconstruction, with FE simulations and a statistical ASED-based fatigue assessment. The framework is systematically evaluated across different strut orientations with respect to the building direction, capturing the effects of surface morphology and defect distribution. By directly comparing predictions obtained from CAD-reconstructed models and CT-based surface meshes, the limits and capabilities of simplified digital twins are critically assessed.

By integrating synthetic as-built geometrical modelling with an energy-based fatigue criterion, the present work advances a scalable framework for fatigue-informed design of AM metamaterials. Although the analysis is conducted at the strut scale, the proposed approach directly supports metamaterial-level predictions by capturing the governing damage mechanisms that control lattice failure initiation. This enables efficient exploration of architectural design spaces, manufacturing parameters, and defect sensitivity without reliance on exhaustive CT-based characterization. More broadly, the framework contributes toward physically grounded digital twins for mechanical metamaterials, bridging architected geometry, manufacturing variability, and fatigue performance within a unified and computationally tractable design methodology.

## Results

**Fig. 1:**
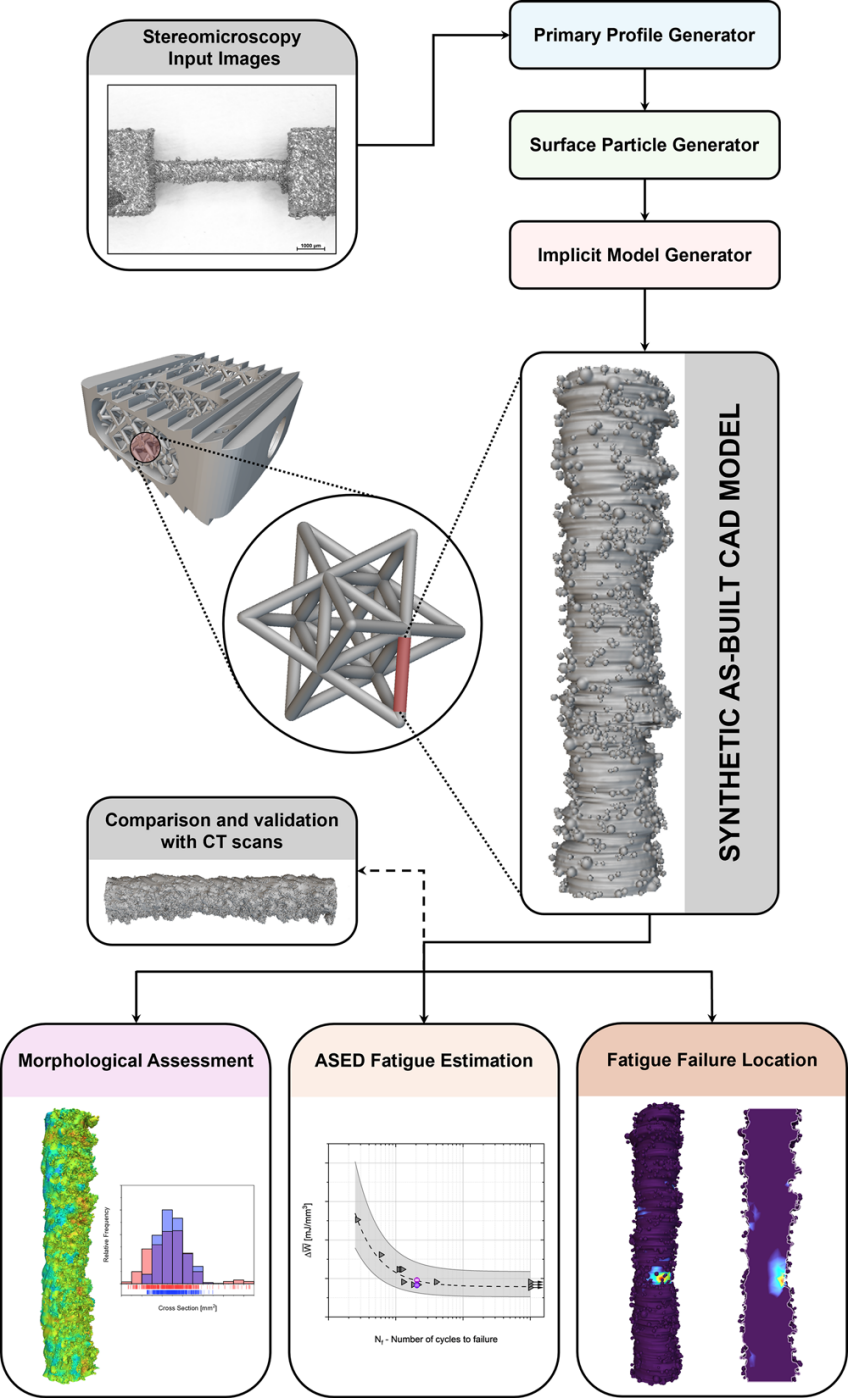
Schematic representation of the proposed workflow for generating synthetic as-built CAD models of miniaturized L-PBF struts, the smallest repeating units of strut-based architected cellular materials. Multi-view stereo-optical images, the framework’s input, are processed through the Primary Profile Generator, Surface Particle Generator, and Implicit Model Generator modules to obtain a 3D synthetic as-built CAD. Surface meshes derived from CT reconstructions are used as reference for geometric validation. The synthetic CAD models are subsequently employed for morphological assessment, statistical ASED-based fatigue life estimation, and fatigue failure location prediction.

Figure 1 schematically illustrates the overall framework adopted in this work for the generation and fatigue assessment of CAD-based digital twins of L-PBF Ti-6Al-4V thin struts, which represent the fundamental repeating units of strut-based architected cellular materials. The framework is applied to struts manufactured at four different build orientations with respect to the build plate (90°, 45°, 15°, and 0°).

Starting from multi-view stereomicroscopy images, each strut geometry is reconstructed through a sequence of dedicated modules, namely the Primary Profile Generator (PPG), Surface Particle Generator (SPG), and Implicit Model Generator (IMG), yielding a 3D synthetic as-built CAD model that explicitly accounts for orientation-dependent surface morphology and partially fused powder particles. The synthetic as-built CAD geometry is then validated against its CT counterpart (a surface mesh derived from metrological CT reconstruction) and subsequently employed as input for the numerical and statistical fatigue analyses.

The framework outputs are organized into three complementary analyses, which also define the structure of this section. First, a morphological assessment quantifies the geometric agreement between synthetic CAD models and CT-based surface meshes in terms of surface deviations and cross-sectional variability. Second, a statistical ASED-based approach is applied to evaluate the fatigue response of the reconstructed struts and to compare fatigue life predictions obtained from synthetic and CT-derived geometries. Finally, the spatial distribution of fatigue-critical ASED-critical locations is analysed to assess the capability of the framework to localize experimentally observed failure sites.

### Morphological characterization

**Fig. 2:**
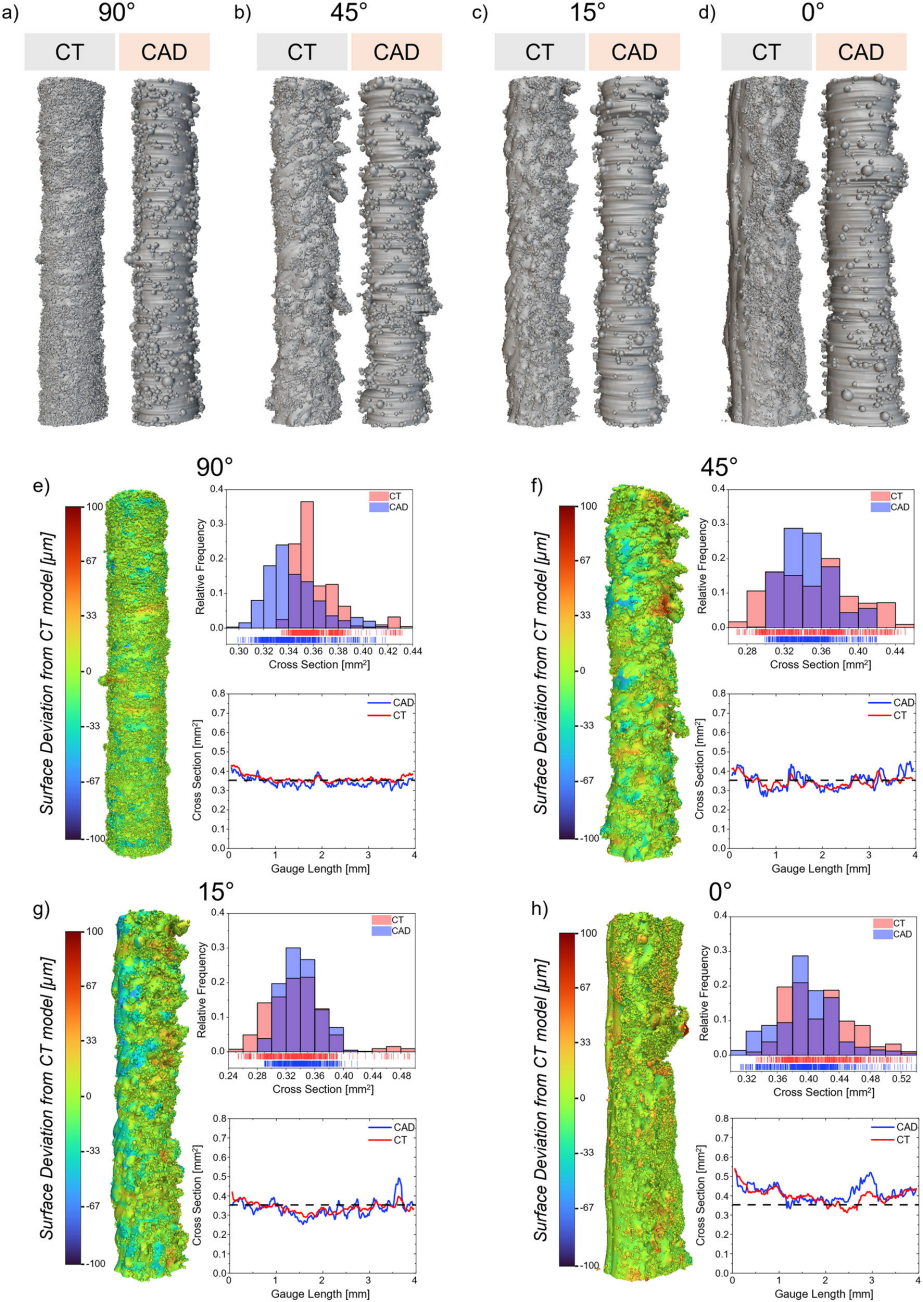
Morphological characterization of synthetic as-built CAD models and comparision with CT-reconstructed struts. **a**–**d** 3D comparison of CT-reconstructed struts (grey) and synthetic CAD models (orange) for vertical (90°), inclined (45°, 15°), and horizontal (0°) build orientations. **e**–**h** Quantitative geometric analysis for each build angle comprising (left) surface deviation plots mapped over CT data showing local geometric discrepancies between synthetic CAD models and CT surface meshes with colour scale ranging from −100 μm (blue, CAD underestimation) to + 100 μm (red, CAD overestimation), (top right) cross-sectional area distribution histograms comparing CAD (light red) and CT (blue) models, and (bottom right) axial evolution of cross-sectional area along the 4 mm gauge length with CAD model (red solid line), CT model (blue solid line), and nominal geometry (black dashed line).

Figure 2 presents a comprehensive morphological assessment of the synthetic CAD models against their CT counterparts across the four investigated build angles (90°, 45°, 15°, 0°), combining qualitative visual comparison (Fig. 2a–d) with quantitative geometric analyses (Fig. 2e–h). The qualitative examination reveals close visual agreement between the CT-based reconstructions and the synthetic as-built CAD-generated geometries, with the latter being able to properly capture the complex as-built surface morphology characteristic of L-PBF thin struts, particularly at the downskin of inclined specimens. Nonetheless, it is worth noting that the synthetic as-built CAD models exhibit evident ridges oriented orthogonally to the specimen’s axial axis, which are ascribable to their elliptical cross-sectional reconstruction and are not visible in the CT-based models. Conversely, the CT reconstructions display milder ridges inclined according to the building angle, particularly visible on the upskin of low-angle specimens (15° and 0°). Surface deviation maps, in Fig. 2e–h, demonstrate that geometric discrepancies between synthetic CAD models and CT-based surface meshes predominantly fall within the ±33 μm range, corresponding to the D_50_ of the feedstock powder, with only localized regions exhibiting more pronounced deviations. Negative surface deviations of the synthetic CAD models relative to the CT strut meshes are observed on the upskin of specimens manufactured at 45° and 15°, while positive deviations characterize the downskin of tilted and horizontal specimens, corresponding to regions where parasitic masses and dross formations are prevalent. Cross-sectional area distributions extracted from both geometries exhibit good overall agreement, with histograms in Fig. 2e–h showing comparable distributions, as also shown by the mean values and dispersion across all build angles reported in Table 1.

**Table 1:** Mean and standard deviation values of the cross-sectional area, calculated along the strut axis and expressed in mm^2^, for the as-built CAD models and CT-based surface meshes for the four building orientations

	Cross Section [mm^2^]											
	90°			45°			15°			0°		
**As-built CAD**	0.343 ± 0.022			0.352 ± 0.042			0.332 ± 0.041			0.414 ± 0.042		
**CT-scan**	0.361 ± 0.019			0.346 ± 0.026			0.339 ± 0.024			0.398 ± 0.039		
**Relative error [%]**	−4.9			1.8			−1.2			3.9		
	**Areal Surface Descriptors [µm]**											
	**90°**			**45°**			**15°**			**0°**		
	***Sa***	***Sv***	***S5v***	***Sa***	***Sv***	***S5v***	***Sa***	***Sv***	***S5v***	***Sa***	***Sv***	***S5v***
**As-built CAD**	12.0	55.4	49.5	16.7	101.8	89.7	16.6	112.7	88.5	12.6	134.2	102.5
**CT-scan**	8.8	66.0	51.8	16.0	129.7	96.6	17.1	116.2	108.6	20.3	222.5	161.7
**Relative error [%]**	36.4	−16.1	−4.4	4.4	−21.5	−7.1	−2.9	−3.0	−18.5	−37.9	-39.7	−36.6

Relative error in % is defined as the difference between the as-built CAD models and the CT surface meshes, normalized with respect to the CT surface data. Areal surface descriptors, namely the arithmetic mean height, Sa, the maximum valley depth, Sv, and five-point pit height, S5v, are reported, in µm, for the synthetic as-built CAD model and CT-based meshes for the four building orientations. Relative error in % is defined as the difference between the as-built CAD models and the CT surface meshes, normalized with respect to the CT surface data.

The plots depicting cross-sectional area evolution along the gauge length (Fig. 2e–h) reveal distinct behaviour depending on build orientation. For the vertical orientation (90°), the synthetic CAD model exhibits systematic underestimation throughout the entire gauge length, with mean values of 0.343 ± 0.022 mm² (CAD) versus 0.361 ± 0.019 mm² (CT), corresponding to a -4.9% relative error. In contrast, inclined and horizontal orientations demonstrate a different pattern, whereby synthetic as-built CAD models systematically produce higher peak values compared to their CT counterparts, particularly in regions affected by parasitic mass formation. This behaviour is attributed to the elliptical cross-sectional assumption employed in the generative framework, which tends to place additional material in the re-entrant portions of parasitic masses, as particularly evident in the 45° specimen comparison (Fig. 2b). These orientations show improved overall correspondence, with relative errors of +1.8% at 45° (0.352 ± 0.042 mm² CAD vs 0.346 ± 0.026 mm² CT), −1.2% at 15° (0.332 ± 0.041 mm² CAD vs 0.339 ± 0.024 mm² CT), and +3.9% at 0° (0.414 ± 0.042 mm² CAD vs 0.398 ± 0.039 mm² CT). CT-based and synthetic CAD models exhibit increasingly pronounced peaks and valleys with decreasing build angle, accompanied by larger fluctuations from the nominal profile (black dashed lines), reflecting the more severe L-PBF process-induced defects characteristic of low-angle builds^17–19^. Despite local deviations also reaching ±50 to ±80 μm in some defect-affected regions, the axial locations of these critical geometric features are consistently well-identified in both representations. To quantitatively validate the morphological agreement between the two models, the two one-sided tests (TOST) procedure was employed on equivalent diameter to assess statistical equivalence within a ± 30 μm (D_50_ of the feedstock powder) margin (*α* = 0.05). All build angles demonstrated equivalence (*p* < 0.001), with 90% confidence intervals of [14.6, 19.5] μm for 90°, [−10.6, 0.0] μm for 45°, [−0.4, 9.7] μm for 15°, and [−20.0, −7.9] μm for 0°, all falling below or around the D_10_ (15 μm) of the feedstock powder, thus validating the morphological fidelity of the proposed synthetic as-built CAD models.

Table 1 additionally reports three areal surface descriptors computed on the unfolded 3D surfaces: the arithmetic mean height, *Sa*, evaluated on the S-L surface to isolate particle-scale morphological features, and two fatigue-relevant valley depth descriptors: the maximum valley depth, *Sv*, and the five-point pit height, *S5v*, both extracted from the S-F surface. *Sa* agreement between synthetic as-built CAD and CT-reconstructed models is orientation-dependent: the 45° and 15° reconstructions reproduce the CT reference within 5% and 3%, respectively, whereas larger deviations arise at the two extreme orientations. At 90°, the overestimation can be attributed to the circumferential ridging introduced by the elliptical cross-sectional assumption, whose contribution to *Sa* is proportionally amplified on the smoothest surface in the dataset. Conversely, at 0° the underestimation is ascribable to the multiple layers of partially fused powder particles adherent to the CT-reconstructed downskin (Fig. 2d), whose contribution to the surface texture is only partially captured by the synthetic model due to shadowing effects inherent to 2D stereomicroscopy images, which cannot fully resolve particles occluded by the outermost layer.

At the 90°, 45°, and 15° orientations, the absolute discrepancy in the single deepest valley *Sv* between the synthetic as-built CAD and CT-reconstructed models falls within the characteristic particle size range of the feedstock powder, indicating satisfactory agreement given the inherent resolution and reconstruction constraints of the stereomicroscopy-based approach (i.e., shadowing effects and optical occlusions) relative to a high-resolution volumetric reconstruction. These limitations become particularly pronounced for the 0° orientation ( − 39.7%), where the aforementioned multiple layers of partially fused particles on the downskin generate deeply re-entrant valleys whose full depth cannot be resolved by the stereomicroscopy-based reconstruction. The five-point pit height *S5v* reveals a monotonically increasing underestimation from −4.4% at 90° to −7.1% at 45°, −18.5% at 15°, and −36.6% at 0°, further highlighting the orientation dependence of the valley detection accuracy. At 0°, the discrepancy is less pronounced than for *Sv*, suggesting that while the deepest re-entrant valley cannot be fully captured beyond a certain depth, this effect is partially mitigated when a broader population of valleys is considered.

### ASED-based fatigue estimation

**Fig. 3:**
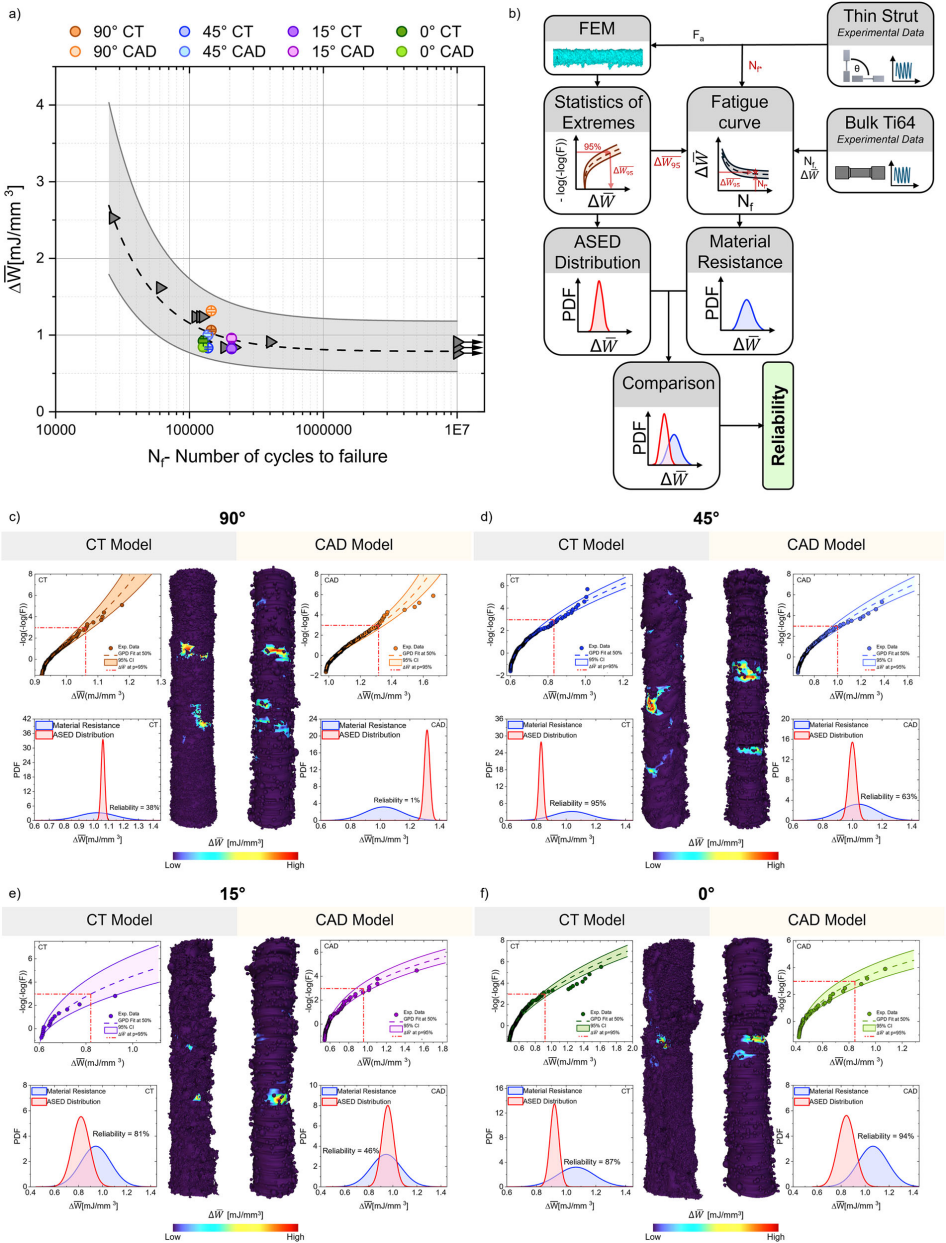
ASED-based fatigue estimation of the CT-reconstructed and synthetic as-built CAD models. **a** The $\Delta{\bar{{{W}}}}-{{\rm{N}}}_{{\rm{f}}}$ chart superimposes the fatigue predictions of the thin-strut specimens geometrical reconstructions based on micro-CT and synthetic CAD models on the reference curve for Ti6Al4V components. **b** Framework for the experimental-numerical analysis of thin-strut fatigue resistance. **c**–**f** Statistical analysis of the average strain energy density critical locations for the estimation of the fatigue resistance. A comparison of the results obtained for the micro-CT based geometrical reconstruction and of the synthetic CAD geometries is performed and the results are analysed in term of reliability of the component against fatigue failure.

The framework employed to estimate the fatigue behaviour of thin strut specimens is schematized in Fig. 3b and combines computational and experimental analysis, leveraging on a statistical application of the ASED local energetic approach^26^. A detailed description of the finite element set-up is provided in *Supplementary Information A* (Figure S1 and Table S1).

Within this approach, fatigue failure is assumed to occur when the amount of elastic energy stored in a critical component location overcomes the fatigue resistance identified by a material-specific $\Delta \bar{W}-{N}_{f}$ reference curve. The latter was derived from the experimental Whöler curve of bulk L-PBF Ti-6Al4-V specimens reported in ref. ^27^, as explained in greater detail in *Section Fatigue Estimation Routine (FER)*. Experimental data from the thin strut fatigue tests were also employed to inform the FE model of both the CT-based and synthetic as-built CAD models by means of the experimental amplitude force, *F*_a_ (see *Section Experimental Fatigue Tests)*.

Although CT-based geometrical reconstruction represents the current gold standard for non-destructive characterization of AM components, its finite spatial resolution may lead to a false or imperfect representation of the surface features critical for fatigue crack initiation. Moreover, as proven by ref. ^28^, even higher-fidelity geometrical reconstructions, such those obtained via synchrotron tomography, do not guarantee the deterministic identification of the fatigue crack initiation sites.

Therefore, a non-deterministic yet statistical approach^24^ is needed, able to comply with possible reconstruction errors and to quantify the associated variation in the local loading conditions. The benefit of a statistical approach for fatigue life estimation is twofold: as discussed, it can account for geometrical imperfect reconstructions, while it can also account for the experimental variability in material resistance^29^. The combination of these two factors, represented as random variables, can be leveraged to provide the reliability of the component against fatigue failure.

Accordingly, a statistical ASED-based approach was employed, as described in detail in *Section Fatigue Estimation Routine (FER)*. This approach is based on the Peak over Threshold (POT) theory, whereby only the most energetically critical locations are considered relevant for fatigue failure^24^. In each distribution, the ASED value ${\Delta}{\bar{W}}_{{95}{\%}}$ associated with the 95% percentile of the distribution, is selected as a threshold: the locations where the ASED is found above $\Delta {\bar{W}}_{95 \% }$ are identified as possible hot-spot able to trigger fatigue failure. The choice of this quantile is supported by the sensitivity analysis reported in *Supplementary Information B* (Figure S2) and in line with^22^. The defined 95% threshold is also correlated with a confidence interval (CI) set at 95% in agreement with^30^. The numerical $\Delta {\bar{W}}_{95 \% }$ values and the confidence intervals are summarized in Table 2, along with the relative error (in %) between the CT-based and synthetic CAD-based models.

**Table 2:** - ${\bar{\rm{W}}}^{*}$ threshold ASED values for the POT approach; ${\rm{\lambda }},{\rm{\delta }},{\rm{\gamma }}$ are fitting parameters for the GPD; ASED values at the 95% quantile ($\Delta{\bar{{\rm{W}}}}_{95 \% }$) in mJ/mm³ and 95% confidence intervals (CI) for as-built CAD and CT-based models across the four building orientations; N_f*_ experimentally driven number of cycles to failure associated at each $\Delta {\bar{{\rm{W}}}}_{95 \% }$ value

Geometry	$\left[\frac{{mJ}}{{{mm}}^{3}}\right]$	GDP Parameters			$\left[\frac{{mJ}}{{{mm}}^{3}}\right]$	[%]	$\left[\frac{{mJ}}{{{mm}}^{3}}\right]$	${N}_{{f}^{* }}$
	${\mathrm{\varDelta W}}^{* }$	${\rm{\lambda }}$	${\rm{\delta }}$	${\rm{\gamma }}$	${\Delta}{\bar{W}}_{95 \% }$	Relative error	CI	
**CT - 90°**	0.92	0.92	0.05	−0.09	1.06	+24.5	[1.05, 1.07]	145,427
**CAD - 90°**	0.98	0.98	0.14	−0.14	1.32		[1.30, 1.33]	
**CT - 45°**	0.60	0.60	0.06	0.14	0.83	+20.5	[0.81, 0.84]	137,582
**CAD - 45°**	0.66	0.66	0.10	0.10	1.00		[0.98, 1.03]	
**CT - 15°**	0.56	0.56	0.05	0.22	0.82	+17.1	[0.78, 0.89]	206,330
**CAD - 15°**	0.54	0.54	0.08	0.31	0.96		[0.92, 1.00]	
**CT - 0°**	0.48	0.48	0.12	0.15	0.92	−8.7	[0.89, 0.95]	126,853
**CAD - 0°**	0.42	0.42	0.08	0.33	0.84		[0.79, 0.91]	

Relative error in % is measured as the difference between the as-built CAD and CT-based models normalized over the CT-based model.

Once measured the $\Delta {\bar{W}}_{95 \% }$, the experimentally derived number of cycles to failure (${N}_{{f}^{* }}$ reported in Table 2) of each tested strut is used to locate the corresponding $\Delta {\bar{W}}_{95 \% }$ value on the $\Delta \bar{W}-{N}_{f}$ fatigue reference curve reported in Fig. 3a, where the predicted $\Delta {\bar{W}}_{95 \% }$ values with their 95% CI (error bars) are shown for both the CT-based (dark colour labels) and the as-built synthetic CAD struts (light colour labels).

For the CT-based models, all ${\bar{W}}_{95 \% }$ predictions fall within the ±1.5 scatter bands of the bulk Ti-6Al-4V reference curve, confirming that the ASED criterion successfully captures fatigue-critical stress concentrations in miniaturized L-PBF struts despite orientation-dependent surface morphology variations. The synthetic CAD models exhibit systematic deviations from CT predictions, however still falling within the ±1.5 scatter bands of the bulk Ti-6Al-4V reference curve. For 90°, 45°, and 15° strut’s orientations, the synthetic as-built CAD model consistently overestimates $\Delta {\bar{W}}_{95 \% }$, with the vertical orientation presenting the maximum discrepancy. The horizontal orientation represents instead the sole case of underestimation of the $\Delta {\bar{W}}_{95 \% }$. Notably, all CAD predictions remain within the reference curve scatter bands despite these deviations, indicating that the proposed CAD-based reconstruction captures fatigue-critical morphological features sufficiently accurately for fatigue life estimation purposes.

Figure 3e–h collects the results of the above-mentioned statistical approach. It is found a clear consistency between the generalized pareto distributions (GPD) of the ASED results of CT-based and synthetic CAD models. The populations of the critical ASED defects are consistent among the different geometrical representations and consistently fit by the proposed distribution. Table 2 collects the parameters for the statistical analysis as presented in *Section Fatigue Estimation Routine (FER)*. A graphical comparison between the ASED distribution of the CT-based and the synthetic CAD geometrical reconstructions shows an overall agreement among each building orientation’s pair. A more in-depth analysis in this matter is carried out in *Section Fatigue Failure Location*.

There is a clear trend in the reliability of both CT-based and synthetic CAD models: the 0°, 15°, and 45° specimens display a reliability close or above 50% for both geometrical representations. There is therefore consistency between micro-CT geometrical reconstructions and synthetic CAD fatigue life estimations. Both are statistically underestimating the loading conditions that produced fatigue failure. In contrast, the 90° specimen is characterized by a reliability below 50% and therefore the represented loading condition is statistically considered more critical to fatigue failure.

Furthermore, these reliability trends directly reflect the morphological discrepancies between CT-based and synthetic CAD geometries quantified in Table 1. The pronounced reliability drop observed for the 90° CAD specimens can be ascribed to the systematic underestimation of effective cross-sectional area relative to CT reconstructions, which reduces the overall strut’s load-bearing area, hence yielding a less conservative fatigue prediction. For the 0° configuration, synthetic CAD models locally overestimate the load-bearing area, particularly at pronounced surface protrusions surrounding the ASED-critical region, producing localized stress-mitigating effects that result in lower $\Delta {\bar{W}}_{95 \% }$ and higher reliability indices compared to CT-based models. The 15° and 45° orientations represent intermediate cases: milder geometric discrepancies along the gauge length combine with moderately larger effective cross-sections in the CT reconstructions within dominant failure regions, consistently explaining the slightly higher reliability values obtained for synthetic CAD models at these build angles. It is however worth noting that $7/8$ of the reliability indexes fall inside a $2\sigma$ dispersion, highlighting the suitability of the proposed fatigue prediction approach.

### Fatigue failure location

**Fig. 4:**
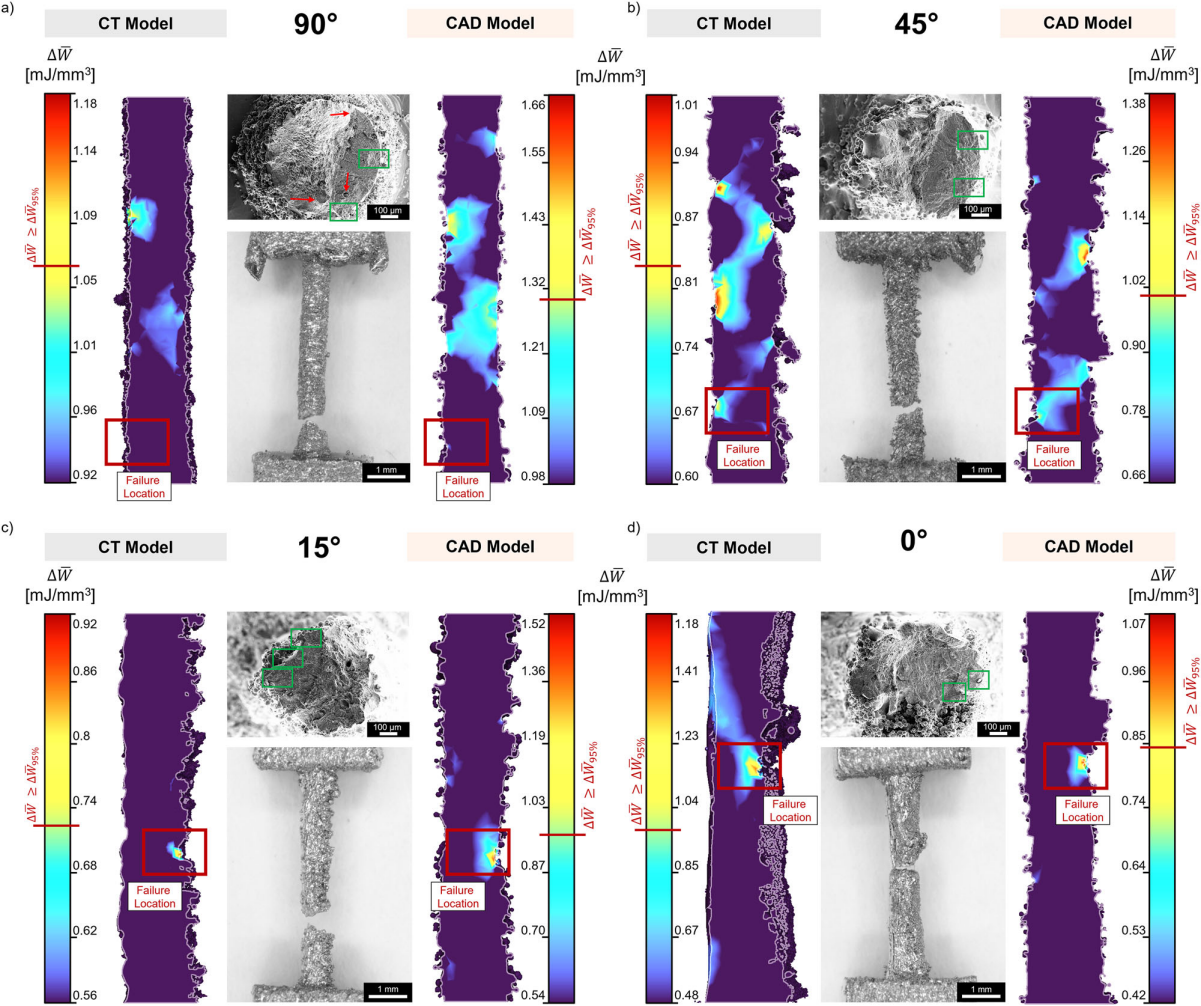
ASED-based prediction of fatigue failure locations in synthetic as-built CAD models and CT-reconstructed struts. **a**–**d** Fatigue failure location prediction through ASED analysis for vertical (90°), inclined (45°, 15°), and horizontal (0°) build orientations. Each panel presents cross-sectional ASED distributions for the CT-based model (left) and synthetic as-built CAD model (right), with colormaps ranging from the POT ASED value to the maximum ASED. The red horizontal line on each colour bar legend marks the 95% quantile threshold (${\Delta \bar{{\rm{W}}}}_{95 \% }$). Red boxes on ASED distributions maps denote the experimentally observed failure locations. Stereomicroscopy images of failed specimens are positioned centrally between each ASED distribution pair, with red boxes indicating the fracture sites. The scale bar for stereomicroscopy images is set to 1 mm. SEM fractographic analyses (top) show failure initiation sites with green boxes highlighting surface micronotches formed by partially molten particles and red arrows (90° only) indicating subsurface porosity contributing to crack propagation. The scale bar for SEM images is set to 100 μm.

Figure 4 shows the cross-sectional views of the ASED colormap distributions for both CT-based and synthetic as-built CAD models across all four build orientations. The colormap scaling ranges from the POT values (reported in Table 2) to the maximum detected ASED values, with the red horizontal line on each legend marking the ${\Delta \bar{W}}_{95 \% }$. As previously discussed, all regions exhibiting ASED values exceeding this threshold represent statistically equivalent potential fatigue initiation sites. The experimentally observed fracture locations are documented through stereomicroscopy images and indicated by red boxes overlaid on each cross-sectional view of the ASED distribution maps. Corresponding scanning electron microscopy (SEM) fracture surfaces of each fatigue-failed specimen are also reported in each panel (Fig. 4a–d). The computational ASED framework successfully captures the experimental failure location in three out of four build orientations, specifically at 45°, 15°, and 0°; conversely, the experimentally observed failure location of the 90° strut fails to be captured neither by the CT nor the synthetic CAD model.

For the 45° inclined orientation (Fig. 4b), ASED values above the ${\Delta \bar{W}}_{95 \% }$ threshold appear at the experimental failure location in both CT and CAD models. The stereomicroscopy image reveals that fracture initiation occurred at a micro-notch on the upskin, with the failure propagating diagonally upward toward a pronounced surface protrusion located on the strut’s downskin. This diagonal propagation is directly reflected in the ASED distributions of both synthetic CAD and CT models, which exhibit an elongated ASED concentration zone extending from the upskin “killer” micro-notch toward this downskin protrusion, demonstrating an interesting spatial correspondence between the computational strain energy localization and the observed failure path. Beyond this primary failure region, both models additionally identify multiple discrete zones along the gauge length where ASED values exceed the critical threshold, several of which register higher magnitudes than those observed at the actual fracture site. The spatial positioning of these ASED regions demonstrates reasonable correspondence between the two modelling approaches, albeit the synthetic CAD model consistently exhibits attenuated ASED magnitudes within several of these zones relative to its CT counterpart.

The 15° and 0° orientations (Fig. 4c, d) both display a strong correspondence between the CT and synthetic CAD predictions, with ASED localizations occurring at identical spatial coordinates along the gauge length, precisely matching the reported experimental fracture locations. Unlike the 45° case, the stereomicroscopy images for these orientations reveal failure originating from a prominent macro-notch situated on the downskin surface. The ASED distributions in both models is strongly concentrated around these macro-notch features, with peak magnitudes substantially exceeding the ${\Delta \bar{W}}_{95 \% }$ threshold and exhibiting considerably greater spatial confinement compared to the more diffuse distribution observed at 45°.

In contrast, the vertical 90° orientation (Fig. 4a) represents the sole case where neither computational models are able to identify ASED values above the ${\Delta \bar{W}}_{95 \% }$ threshold at the experimentally driven failure location. Both models nonetheless exhibit comparable ASED distributions at alternative locations along the gauge length, suggesting consistent strain energy localization patterns between the two modelling frameworks despite their inability to capture the actual failure site. This discrepancy therefore warrants consideration of additional failure mechanisms beyond surface geometry-driven stress concentrations. While SEM fractographic analysis confirms that fatigue crack nucleation occurred at surface micro-notches formed by partially molten powder particles, as highlighted by green boxes on the fracture surfaces and consistent with the nucleation mechanism observed across all orientations^19^, the 90° specimen uniquely exhibits internal porosity actively participating in the failure process, specifically during crack propagation stages, as indicated by red arrows in the SEM image reported in Fig. 4a. In brief, fractographic evidence suggests that in 90° struts, subsurface defects can act as secondary crack initiation sites or accelerate propagation in a manner not captured by purely topographic digital twins. This subsurface porosity contribution represents a damage mechanism not captured by the ASED analysis employed in the current framework. Consequently, future iterations of this framework should aim to integrate a stochastic internal pore generator, potentially based on volumetric CT statistics, to account for the competitive failure mechanisms between surface roughness and internal flaws in high-aspect-ratio vertical specimens. For more detailed information on the fracture surface porosity, the reader is addressed to *Supplementary Information C* (Figure S3).

### Framework’s computational time

**Fig. 5:**
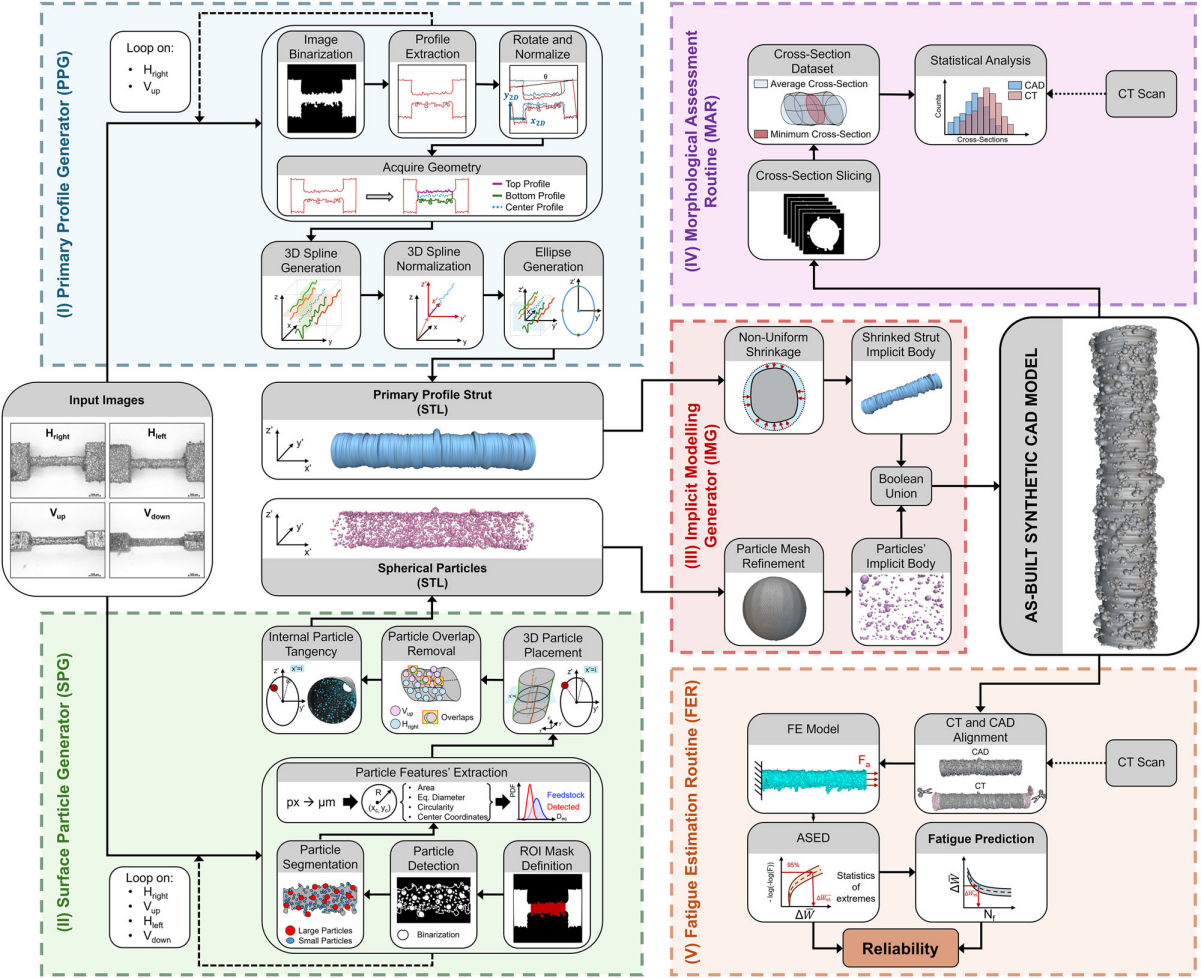
Detailed overview of the framework architecture for the generation, validation, and fatigue assessment of synthetic as-built CAD models of L-PBF Ti-6Al-4V thin struts. The framework is structured into five sequential modules: (I) the Primary Profile Generator (PPG, blue box), (II) the Surface Particle Generator (SPG, green box), (III) the Implicit Model Generator (IMG, red box), (IV) the Morphological Assessment Routine (MAR, purple box), and (V) the Fatigue Estimation Routine (FER, orange box).

Table 3 summarizes the computational time associated with the proposed framework, comparing the end-to-end synthetic as-built CAD workflow with the extended workflow including CT-based validation (see Fig. 5 for a detailed overview of the framework).

**Table 3:** Computational time reported for each processing module in the end-to-end synthetic as-built CAD workflow, the CT-scan validation and in the extended workflow including both the synthetic CAD workflow and the CT-based validation

Task	Computational Time (s)		
	Synthetic As-built CAD	CT-scan Validation	Synthetic CAD + CT-scan
*PPG*	94	–	94
*SPG*	80	–	80
*IMG*	294	–	294
*FER*	1673	1786	3459
*MAR*	51	64	115
**Total**	**2307**	**1850**	**4042**

Total computational times for each workflow are reported in bold.

The synthetic CAD workflow, spanning from stereomicroscopy image processing to fatigue reliability estimation, is completed in approximately 40 min. The generation of the three-dimensional synthetic as-built strut geometry through the PPG, SPG, and IMG constitutes the first stage of the workflow and requires about 8 min, defining the computational cost of constructing a full 3D synthetic as-built CAD model directly from stereomicroscopy images.

The remaining runtime is associated to the Morphological Assessment Routine (MAR) and the Fatigue Estimation Routine (FER). The FER constitutes the dominant contribution to the overall runtime, owing to FE meshing, FE simulation as well as ASED-based fatigue post-processing analysis. These two modules can be run just for the synthetic as-built CAD module or by also including a CT counterpart for validation, increasing the total computational time to approximately 1 h, due to the additional cost primarily associated with CT-based geometry handling and FE analysis.

## Discussion

This work presents a computationally efficient and cost-effective framework for fatigue resistance modelling of AM struts, which represent the fundamental sub-unit elements governing the fatigue behaviour of strut-based metamaterials. The novelty of the proposed approach lies in the generation of functional synthetic as-built CAD digital twins derived exclusively from low-cost stereomicroscopy images, combined with a statistical application of the ASED criterion, specifically tailored to miniaturized L-PBF specimens. The proposed synthetic as-built CAD models are able to retain the principle geometrical features that drive the fatigue damage and are validated against surface meshes derived from CT reconstructions, which currently represent the gold standard non-destructive technique for as-built geometric mechanical characterization in the metal AM field. While such comparison is used here for validation purposes, the proposed framework is conceived as a step toward stand-alone, fatigue-oriented design methodologies, enabling reliability-informed fatigue design optimization of architected components without the need for routine tomographic scanning. It should be noted that the validation was conducted on four specimens manufactured using identical process parameters, differing only in build orientation. Consequently, the framework was not evaluated through multiple replicates of a single orientation, but rather across four orientation-dependent as-built conditions. Since build orientation influences the resulting surface morphology, cross-sectional geometry, and defect population even when the printing parameters remain unchanged, this validation strategy enables assessment under defect scenarios that are mechanically distinct and directly relevant to fatigue behaviour 19. A more comprehensive statistical assessment of inter-specimen variability will be addressed in future work.

Two key modelling assumptions underpin the proposed framework. First, the as-built strut geometry is reconstructed through orientation-dependent, non-uniformly shrunk elliptical cross-sections informed by stereomicroscopy profiles and surface particle detection. Second, fatigue failure is assumed to be predominantly driven by surface-connected defects, in agreement with extensive experimental fatigue campaigns reported for miniaturized L-PBF Ti-6Al-4V struts tested in the as-built condition^19^. These assumptions intentionally trade geometric completeness for robustness and computational efficiency, that supports rapid generation of synthetic as-built CAD models and their use in time efficient FE analyses and statistical fatigue assessments without reliance on volumetric imaging.

Within this scope, the morphological analyses demonstrate that the synthetic CAD models efficiently capture the dominant manufacturing-induced features across all investigated build orientations. In particular, the framework reproduces the axial evolution of cross-sectional variability, the formation of parasitic masses on the downskin of inclined and horizontal struts, and the amplification of surface irregularities at low build angles. The statistical equivalence demonstrated by the TOST test confirms that the synthetic models preserve the overall geometric fidelity required for fatigue-relevant assessments. Local geometric discrepancies with respect to CT-based reconstructions are nonetheless observed, primarily associated with the 2D image-based elliptical cross-sectional reconstruction, and are further reflected in the orientation-dependent deviations of the valley depth descriptors *Sv* and *S5v*. These discrepancies do not, however, translate proportionally into fatigue prediction errors, due to the fundamental difference between point-wise surface metrics and volumetric energy-based criteria. Crucially, the axial locations of critical geometric features, *viz*. micro- or macro-notches at valleys or particle protrusions, are consistently identified by the synthetic as-built CAD models, which is the essential prerequisite for reliable ASED-based localization of fatigue-critical regions.

As a matter of fact, solely relying on the depth of the single deepest or the five deepest valleys does not account for the mutual stress-field interaction among adjacent surface features, which can either amplify or shield the local driving force depending on their relative spacing and geometry. The volumetric ASED formulation can instead overcome this limitation by integrating the strain energy density over a physically motivated control volume, thereby naturally accounting for the combined effect of multiple neighbouring notches and their synergistic contribution to the local stress state. In this sense, the proposed digital twin is purposely designed to serve as a fatigue prediction tool rather than as a metrologically exhaustive replica of the CT-reconstructed surface, effectively translating geometric defectiveness into fatigue-relevant metrics in a physically grounded manner.

Across most build orientations, the synthetic CAD models yield ASED predictions that are biased toward safety relative to CT-based reconstructions. This behaviour is particularly advantageous from a design perspective, as it allows the framework to be employed for geometry optimization and defect-tolerant design without underestimating fatigue risk. The inclusion of reliability as an explicit output further strengthens the framework’s design relevance, as it enables fatigue performance to be interpreted probabilistically rather than deterministically^24,29^. Unlike traditional deterministic approaches based on safety factors, reliability indices explicitly capture the probability of survival under prescribed loading conditions, thereby offering a more rigorous basis for design decisions where competing failure modes and geometric variability must be balanced against performance requirements. Probabilistic approaches prove particularly valuable in AM contexts, where the inherently stochastic nature of fatigue damage is amplified by process-induced geometric deviations and internal defect populations that introduce additional sources of uncertainty beyond conventional material scatter. Moreover, reliability-based frameworks allow overcoming technological limitations inherent to state-of-the-art computational and metrological resources, which currently provide no efficient means to deterministically estimate fatigue resistance and failure location with both accuracy and precision in geometrically complex AM components. These combined challenges necessitate statistical frameworks capable of propagating multiple uncertainty sources into actionable design metrics.

Beyond fatigue life estimation, the framework demonstrates a strong capability to identify experimentally observed failure locations among the statistically critical ASED regions. The predicted failure sites consistently correspond to surface-driven stress raisers, supporting the underlying assumption that fatigue damage in miniaturized struts is governed by local surface morphology^19^. Importantly, the approach captures failure mechanisms associated with both macroscopic geometric deviations, such as downskin macro-notches, and micro-scale surface irregularities arising from heterogeneous melt-track solidification, as observed in 45° inclined specimens.

The limitations of the proposed framework emerge naturally from its underlying assumptions. The use of elliptical cross-sections introduces a systematic tendency to include additional material in re-entrant regions of surface protrusions, which can mitigate local stress concentrations and hence possibly explain the more conservative ASED predictions of the synthetic as-built CAD models. The ridge patterns introduced by the elliptical cross-section reconstruction are inherently aligned with the strut axis, whereas CT-based reconstructions reveal surface ridges’ inclination governed by printing orientation, scanning strategy and melt-track direction. Addressing this limitation will require future extensions of the framework toward tilted or directionally informed cross-sectional reconstructions to achieve a more spatially accurate stress distribution.

A second limitation concerns the treatment of internal porosity. While surface defects dominate fatigue crack initiation in most investigated cases, experimental evidence shows that porosity can actively participate in crack propagation for specific orientations and loading conditions^19^. In particular, the relative dominance of surface roughness versus internal flaws is governed by the interplay between build orientation, which controls both the severity of surface stress raisers and the volumetric porosity level, and the stress ratio. As the compressive portion of the fatigue cycle increases, crack closure progressively shields surface defects while the crack-driving role of subsurface pores is amplified. As porosity information cannot be retrieved from stereomicroscopy images, it is intentionally excluded from the present framework. Consequently, the proposed synthetic as-built CAD model should be regarded as a surface-topography digital twin, whose domain of validity is confined to fatigue regimes in which surface defects remain the dominant crack-driving features. The mismatch between the predicted and experimental failure location for the 90° specimen in this study can be explained by the synergistic interaction of surface and subsurface defects, consistently with the experimental findings reported in^19^, where vertical struts under a fully reversible or predominantly compressive fatigue regimes were shown to be more susceptible to this combined damage mechanism, which the present surface-topography twin is not designed to capture. Extending the framework to include porosity will require a hybrid strategy coupling the present stereomicroscopy-based surface reconstruction with a statistically plausible pore population generated, for instance, through a statistics-of-extremes treatment of CT-derived porosity data, hence accepting a shift from deterministic digital twins toward statistically representative ones. In parallel, a regime-transition metric, based on surface roughness descriptors, orientation-dependent volumetric porosity, and the applied stress ratio, should be developed to discriminate surface-dominated, mixed, and porosity-dominated fatigue conditions and to select the appropriate modelling route accordingly. Despite these limitations, the proposed framework establishes a scalable route toward fatigue-informed digital twins for the design of architected metamaterials that does not rely on high-resolution CT data. By prioritizing functional accuracy over geometric exhaustiveness, the proposed approach enables the generation of as-built CAD models suitable for design and modelling stages. Extending this strategy to ensembles of struts and lattice junctions will allow the construction of as-built CAD metamaterials that can inform fatigue-driven optimization at the component scale. Transferring the methodology to lattice-level components will require a multiscale strategy. Computationally efficient reduced-order models, such as compensated beam formulations^31^, could first be used to identify potentially critical regions within the global lattice component, which could then be re-analysed as detailed as-built sub-models according to the workflow proposed herein. This would confine the high-fidelity ASED assessment to localized domains of interest, thereby limiting the computational burden associated with the analysis of the full lattice structure. A complementary or alternative route could rely on the homogenization of imperfect unit cells^32^. In this way, the effective response of the lattice could retain the influence of strut-level geometric defectiveness without requiring the explicit reconstruction of each individual strut member. Lattice junctions, which are recognized as fatigue-critical regions^33,34^, could, in principle, also be incorporated into the proposed digital-twin CAD framework. However, due to their geometric complexity, data-driven reconstruction strategies trained on hybrid datasets combining high-resolution CT acquisitions with cost-effective synthetic as-built strut geometries may provide a more scalable approach than stereo-optical based synthetic CAD digital twins.

## Methods

### Specimen manufacturing and input image acquisition

Miniaturized dog-bone specimens were designed to represent isolated lattice thin struts, with a nominal thickness of 0.67 mm, a gauge length of 2.4 mm, and an overall grip-to-grip axial length of 15 mm. For additional information of the specimen’s design, the reader is addressed to^17,19^.

Specimens were fabricated by L-PBF using Ti-6Al-4V Grade 5 powder (15–45 µm, O₂ < 0.2%) on an EOS M290 system (400 W laser, 60 µm layer thickness) at Lincotek Medical facilities (Trento, Italy). Process parameters were chosen to reflect industrial manufacturing conditions. Post-build heat treatment above 800 °C was applied to relieve residual stresses and obtain an α + β lamellar microstructure. One specimen per build orientations (0°, 15°, 45°, and 90° relative to the build plate) was investigated, to intentionally account for orientation-dependent variations in surface morphology and cross-sectional geometry, spanning the full spectrum of process-induced geometric variability characteristic of L-PBF thin struts, from the more homogeneous melt-pool conditions of vertical builds, through the progressive stair-stepping effects of inclined specimens, to the enhanced parasitic mass formation driven by gravitational effects in low-angle and horizontal struts^18–20^. All specimens were tested in the as-built surface condition. Supports were limited to the grip sections, leaving the gauge length unsupported. Samples were removed by electrical discharge machining, and residual supports were mechanically detached.

The methodology proposed in this work is grounded on multi-view stereomicroscopy image acquisition by means of a Nikon SMZ25 stereomicroscope. For each specimen, four images were acquired from orthogonal viewpoints by rotating the specimen of 90° between consecutive acquisitions, yielding views designated as ${H}_{{\rm{right}}}$, ${H}_{{\rm{left}}}$, ${V}_{{\rm{up}}}$ and ${V}_{{\rm{down}}}$ that constitute the input for the synthetic CAD reconstruction pipeline. All images were acquired under identical acquisition settings, a scale of 3.23 µm/px, and using a coaxial illumination system with constant intensity directed normal to the specimen surface.

### X-ray computed tomography

The fabricated specimens were analysed by metrological X-ray micro computed tomography, enabling non-destructive three-dimensional characterization of both external geometries and internal defects^35^. In this work, the four dog-bone specimens built at different orientation angles were scanned using a metrological CT system (Nikon Metrology MCT225) to extract reconstructed volumetric data for geometric assessment and porosity analysis. The system is equipped with a 225 kV microfocus X-ray source and a 16-bit flat panel detector with a $2000\,\times 2000$ pixel matrix, and features high-precision guideways and temperature-controlled cabinet operating at 20 ± 0.5 °C. Given the minute size of the specimens, a voxel size of 3.0 µm was achieved, enabling clear imaging of internal porosities as well as fine surface features, such as partially sintered powder particles.

The main scanning parameters were X-ray tube voltage of 170 kV, tube current of 41 µA, and 2000 single-frame projections acquired with an exposure time of 1400 ms. Three-dimensional reconstructions were subsequently processed using VG Studio MAX (Volume Graphics GmbH, Germany).

A local-adaptive surface determination based on grey value gradients was first applied to obtain an accurate segmentation of the specimen geometry, including regions with local contrast variations caused by surface-attached powder particles. The same reconstructed volumes were then used for porosity analysis, following the local-adaptive segmentation procedure detailed in^36^.

The obtained CT volumetric data were used to extract CT-based surface meshes, which served both as geometric input for finite element analyses and as reference for validating the synthetic as-built CAD digital twin, as well as to provide quantitative porosity information relevant for the interpretation of the observed fatigue and fracture mechanisms.

### Model framework

The computational framework employed in this work follows a sequential pipeline designed to generate synthetic as-built CAD models of L-PBF Ti-6Al-4V thin struts and to evaluate their fatigue response. As previously discussed, and as illustrated in Fig. 5, the proposed workflow comprises five modules: *(i)* the Primary Profile Generator (PPG), *(ii)* Surface Particle Generator (SPG), *(iii)* Implicit Model Generator (IMG), *(iv)* Morphological Assessment Routine (MAR), and *(v)* Fatigue Estimation Routine (FER). The computational framework was implemented in MATLAB R2024b (MathWorks, USA), which served as the supervisory environment for coordinating the five processing modules, data handling, and overall workflow execution. Implicit geometry generation, volumetric meshing, finite element analyses, and post-processing were performed in batch mode via scripted calls to nTop v5.58.3 (nTop, USA) and Ansys Mechanical APDL v2024R1 (ANSYS, USA). The entire pipeline was fully automated, enabling reproducible end-to-end generation, simulation, and analysis. The framework was executed on a desktop workstation equipped with an Intel® Core™ i9-12900KF CPU, an NVIDIA GeForce RTX 3060 GPU, and 126 GB of RAM.

### Primary profile generator (PPG)

The primary strut geometry was retrieved through a profile-based approach in which the stereomicroscopy images underwent binarization *(“Image Binarization”* in Fig. 5) to isolate the strut silhouette, followed by extraction of the specimen’s external borders (*“Profile Extraction”*). To ensure geometric consistency across views and minimize misalignments introduced during acquisition, the extracted profiles were rotated and normalized to define a common local 2D reference system (${x}_{2D},{y}_{2D}$), in which the strut gauge length was aligned with the axial direction ${x}_{2D}$ and the origin was centred at the lowest detectable point on the left grip’s downskin (*“Rotate and Normalize”*). A transition point separating the grip and gauge regions was automatically identified on each normalized 2D profile based on the change in slope at the vertical side of the left grip. The beginning of the region of interest (ROI), corresponding to the gauge length, was determined by applying a fixed axial ${x}_{2D}$ offset of 500 µm from the transition point, while the ROI’s end was identified after 4 mm. Within this region, the strut borders were sampled at discrete positions along the longitudinal ${x}_{2D}$ direction at 6 µm intervals, yielding paired upper and lower boundary coordinates. At each sampled axial location, ${x}_{{2D}_{i}}$, with $i$ spanning the axial extent of the ROI, the local centre was defined as the midpoint between the corresponding upper and lower ${y}_{2D}$ coordinates, thereby generating the centre profile over the gauge length (*“Acquire Geometry”*).

These operations were repeated for a pair of orthogonal stereomicroscopy views ($${H}_{{\rm{right}}}$$ and $${V}_{{\rm{up}}}$$), and the ($${x}_{{2D}_{i}},{y}_{{2D}_{i}})$$ profile coordinates were combined to reconstruct the 3D geometry. A global Cartesian reference system $$\left(x,|,y,|,z\right)$$ was introduced, with the $$x$$ axis aligned with the strut axial direction, hence the $${x}_{2D}$$ of the stereomicroscopy images. The $$y$$ and $$z$$ coordinates were independently retrieved from the two images $${H}_{{\rm{right}}}$$ and $${V}_{{\rm{up}}}$$. The $${y}_{2D}$$ coordinate measured in the horizontal view ($${H}_{{\rm{right}}}$$) was mapped to the global $$y$$-direction, whereas the $${y}_{2D}$$ coordinate measured in the vertical view ($${V}_{{\rm{up}}}$$) to the global $$z$$-direction. This mapping provides 5 sets of 3D points, defined discretely along the gauge length. Each of these sets, extracted from the upper, lower and central profile of the 2D images, was then interpolated independently using a using cubic spline function to generate five continuous splines, namely the upper, lower, left, right and central splines (*“3D Spline Generation”*). To ensure geometric consistency across specimens, a normalized global reference system $$({x}^{{\prime} },{y}^{{\prime} },{z}^{{\prime} })$$ was subsequently defined, as shown in Fig. 5. This system was obtained by rigidly translating all spline coordinates such that the origin coincided with the first point of the centre spline, corresponding to the beginning of the gauge region. Following this normalization, all spline geometries were expressed consistently in the $$({x}^{{\prime} },{y}^{{\prime} },{z}^{{\prime} })$$ (*“3D Spline Normalization”*).

At each sampled ${x}^{{\prime} }$ position, the interpolated spline data were used to define an elliptical cross-section (*“Ellipse Generation”*), whose centre and semi-axes ($a,b$) varied smoothly along the strut. The semi-axis $a$ was defined as half the distance between the upper and lower 3D splines’ points, while the semi-axis $b$ as the half distance between the left and right 3D splines’ points. The assumption of elliptical cross-sections is grounded in the physics of the L-PBF process, which, particularly at inclined low angles, exhibits severe deviations from the circular nominal shape due to stair-case effects and gravitational-induced formation of parasitic masses and drosses on the specimen’s downskin^18,20^. The resulting sequence of elliptical cross-sections provided a continuous 3D representation of the primary strut body surface, which was subsequently discretized into a watertight surface mesh and exported as STL geometry for further processing (*“Primary Profile Strut (STL)”*).

### Surface particle generator (SPG)

In parallel with the PPG, surface-attached particles, representative of partially fused powder, were reconstructed through the SPG operating on the same stereomicroscopy images. The SPG provides an independent geometric description of local surface protrusions, which is later merged with the primary strut geometry during the implicit modelling stage.

Particle detection was performed independently on each stereomicroscopy image within the predefined ROI (*“ROI Definition Mask”*) using a dual-channel, multi-scale Laplacian-of-Gaussian (LoG) blob-detection strategy^37–39^. After a contrast equalization and Gaussian smoothing of the image, LoG responses were computed at two characteristic scale sets: a high-intensity channel targeting fine, bright cluster of particles, binarized via regional maxima and separated using watershed segmentation, and a low-intensity channel targeting larger particles, binarized via response maxima (*“Particle Detection”)*. By imposing geometrical and morphological (size, circularity and solidity) hard-constraints, false-positive detections and overlaps between the two binarized images were resolved and retained particles segmented (*“Particle Segmentation”*). Centroid coordinates, area, equivalent diameter and circularity of each segmented particle were extracted, following a pixel-to-µm conversion, and compared to feedstock particle data (*“Particle Feature Extraction”*). To compensate for projection and truncation effects^40^ prior to 3D particle reconstruction, a size-correction factor based on this statistical comparison was performed.

Three-dimensional particle placement was performed in the same normalized global reference system $\left({x}^{{\prime} },{y}^{{\prime} },{z}^{{\prime} }\right)$ adopted for the primary strut reconstruction, under the assumption of locally elliptical cross-sections. For each detected particle, the particle’s centre (${x}_{{2D}_{c}},\,{y}_{{2D}_{c}})$, extracted from the normalized 2D images, provided the axial $x{\prime}$ coordinate, directly assigned as ${x}^{{\prime} }={x}_{c}$. At the corresponding $x{\prime}$ position, the particle centre was then located on the perimeter of the local elliptical cross-section defined by the interpolated 3D splines. Depending on the stereomicroscopy view, the measured ${y}_{{2D}_{c}}$ coordinate directly provided either the ${y}^{{\prime} }$ or ${z}^{{\prime} }$ coordinate, while the remaining coordinate was computed by enforcing the local ellipse constraint (1):

1 $$\frac{\Delta {y{\prime} }^{2}}{{a}^{2}}+\frac{\Delta {z{\prime} }^{2}}{{b}^{2}}=1$$

where $\Delta y{\prime}$ and $\Delta {z}^{{\prime} }$ denote the offsets of the particle centre from the ellipse centre along the ${y}^{{\prime} }$ and ${z}^{{\prime} }$ directions, respectively (*“3D Particle Placement”*). Particle coordinates reconstructed from the four views were condensed into a single 3D population and overlapped detections of the same physical particle from multiple views were removed (*“Particle Overlap Removal”*).

At this stage, the reconstructed particle centres lie on the perimeter of the local elliptical cross-sections, such that particle geometries are initially positioned as hemispherical protrusions coincident with the primary strut surface. Since the primary profile reconstruction already represents the external envelope of the as-built strut, including the contribution of surface-attached particles, direct superposition would result in non-physical overlap with the strut body. This inconsistency was resolved by imposing an internal tangency condition, whereby particle centres were translated radially inwards until their surfaces became internally tangential to the local elliptical profile (*“Internal Particle Tangency”*). The resulting particle population was then exported as a standalone surface mesh (STL) for subsequent implicit union with the primary strut geometry (*“Spherical Particles (STL)”*).

### Implicit model generator (IMG)

Both the primary profile strut and the spherical particles STL geometries were subsequently imported within an implicit modelling environment (nTop v5.58.3) and converted into implicit bodies. Prior to this conversion, particle meshes were re-meshed and filtered to ensure a uniform surface discretization compatible with implicit-body generation (*“Particle Mesh Refinement”*). The use of implicit representations enables robust Boolean operations and the application of remap fields to control geometric modifications while preserving surface continuity. To reproduce orientation-dependent geometric bias induced by the L-PBF process, a non-uniform shrinkage field was applied to the primary strut implicit body through a remap field function (*“Non-Uniform Shrinkage”*). The shrinkage magnitude varied independently along the lateral ($y{\prime}$) and frontal ($z{\prime}$) directions and was defined as a continuous scalar field based on the local radial distance from the strut centerline. Direction-specific shrinkage of the elliptical cross-sections, based on the particle D_90_ equivalent diameter of each stereomicroscopy image, was performed for the different strut skins (side and upper/lower), and smoothly blended using arctangent-based transition functions to avoid sharp discontinuities in the deformation field. This procedure yielded a shrinkage-modified primary strut implicit body that captures asymmetric material loss and spatially different surface characteristic typical of the as-built state of L-PBF miniaturized components. The shrunk primary strut implicit body was then combined with the particle implicit body through a Boolean union operation, generating a single continuous implicit representation of the as-built conditions of the thin strut, thus obtaining the final sturt model, namely the as-built synthetic CAD model.

### Morphological assessment routine (MAR)

The Morphological Assessment Routine (MAR) was used to extract and compare cross-sectional area from synthetic as-built CAD and CT-based strut geometries, as depicted in Fig. 5. The synthetic implicit model was sliced along the gauge length, and each cross-section was binarized to compute the enclosed area, yielding an axial dataset of cross-sectional areas. The same slice-wise binarization and area extraction procedure was applied to the CT-based strut geometry so to obtain an equivalent cross-sectional dataset from the experimentally measured specimens. The resulting area distributions were then compared and their evolution along the gauge length evaluated. Statistical equivalence between synthetic CAD-generated and CT-derived cross-sectional area distributions was assessed using a two one-sided tests (TOST) procedure, providing a quantitative measure of morphological agreement between the two representations. Areal surface descriptors were computed from the unfolded 3D surfaces of both synthetic as-built CAD and CT-reconstructed models in accordance with ASTM F3624-23^41^. After S-filtering (with nesting index at 0.015 mm) and form removal via a second-order polynomial (F-operator), the resulting S-F surface was used to extract the maximum valley depth, *Sv*, and the five-point pit height, *S5v*, through watershed segmentation (5% Wolf’s pruning). The arithmetic mean height, *Sa*, was evaluated on the S–L surface obtained after additional application of an L-filter (nesting index at 0.8 mm) to the S-F surface to isolate the particle-scale roughness component relevant for morphological comparison. The field and feature parameters were calculated in accordance with ISO 25178-2^42^.

### Fatigue estimation routine (FER)

Following the as-built synthetic CAD model generation, the CT-scan counterpart was also converted into an implicit body and aligned to the CAD model within the same reference system. Then, the CT-based geometry reconstruction of each miniaturised specimen, as well as the synthetic CAD models were meshed and simulated thanks to a linear elastic finite element model (Ansys Mechanical APDL v2024R1 – ANSYS, USA) to reproduce the experimental uniaxial testing conditions. To replicate the experimental set-up, one of the geometry extrema is fixed, while the other one is subjected to the same force amplitude ${F}_{a}$ applied in the fatigue testing as listed in Table 4.

**Table 4:** Experimental force amplitude (*N*) applied to finite element simulations to replicate experimental set-up for each strut's building orientation (indicated in bold)

Force Amplitude - ${F}_{a}$ [$N$]			
**90°**	**45°**	**15°**	**0°**
72	61.5	46	49

In agreement with^24,43^, the critical locations for fatigue failure are identified in the FE model output: peaks of the equivalent Von Mises stress are hot spots where the statistical ASED method for fatigue estimation is applied. Starting from the highest Von Mises stress, the elements contained in a sphere of radius ${R}_{1}=0.152\,{mm}$^24^ centred in the peak stress locations, undergoes the calculation of the ASED as per (2):

2 $$\bar{W}=\,\frac{{\sum }_{i}^{{n}_{e}}{V}_{e}\,{W}_{e}}{{\sum }_{i}^{{n}_{e}}{V}_{e}}$$

where $\bar{W}$ is the ASED, ${n}_{e}$ the number of elements in the selection sphere and ${V}_{e},\,{W}_{e}$ the element volume and the strain energy density, respectively. Once the calculation is completed, these elements are deselected, and the remaining ones undergo the same procedure until the entire component is analysed.

The so-obtained population of ASED point is analysed thanks to the principles of the statistics of extremes to identify the location of possible fatigue failure. In this statistical approach, an asymptotical distribution is proposed to model the behaviour of a given population at its extreme values^44^. The solution of the FE model granted the knowledge of the entire ASED specimen population, and therefore a POT approach^24,30^ is applied. In this method, the population members above a given threshold ${x}\ge {\bar{W}}^{* }$ are considered critical for fatigue failure. The selection of ${\bar{W}}^{* }$ is performed through the identification of the plateau region in the mean excess plot^24,30^. Based on this threshold, a Generalised Pareto Distribution (GPD), as per (3), is fit on each ASED population using the Maximum Likelihood method:

3 $${W}_{\lambda ,\delta ,\gamma }\left(x\right)=\,1-{\left(1+\gamma \frac{x-\lambda }{\delta }\right)}^{-\frac{1}{\gamma }}{\rm{for}}\,\gamma \ne 0$$

where ${W}_{\lambda ,\delta ,\gamma }\left(x\right)$ is the cumulative distribution function of the GPD, $\lambda ,\delta ,\gamma$ are the fitting parameters of the distribution and $x$ is one element of the ASED population. As mentioned in *Section ASED-Based Fatigue Estimation*, only the locations associated with the highest ASED values are considered critical for the fatigue failure and thus the 95% quantile of the GPD distribution ${\Delta \bar{W}}_{95 \% }$ is computed. The confidence intervals $\left[{\Delta \bar{W}}_{{up}},{\Delta \bar{W}}_{{lw}}\right]$ associated with quantile can be computed leveraging a pivotal ${\chi }^{2}$ distribution as per (4):

4 $$\left\{\begin{array}{l}{\Delta{\bar{W}}}_{{up}}=\lambda +\,\frac{2{N}_{s}\,\left({\Delta {\bar{W}}_{95 \%}}-\lambda \right)}{{\chi }^{2}\left(\frac{1+\alpha }{2},2{N}_{s}\right)}\\ {\Delta \bar{W}}_{{lw}}=\lambda +\,\frac{2{N}_{s}\,\left({\Delta {\bar{W}}_{95 \%}}-\lambda \right)}{{\chi }^{2}\left(\frac{1-\alpha }{2},2{N}_{s}\right)}\end{array}\right.$$

Where ${N}_{s}$ is the number of population individuals and $\alpha$ the confidence interval desired, fixed at 95%^30^. The selection of the 95% quantile of the distribution as index for the determination of critical locations for fatigue failure denotes a trade-off between the requirement of selecting only the most critical locations and the requirement to provide enough population individuals in the ASED analysis. The fatigue life prediction of as-built synthetic CAD models and their CT counterparts were assessed leveraging on a reference curve of L-PBF Ti-6Al-4V. The latter was obtained exploiting experimental data available in^27^ from bulk unnotched specimens manufactured with the L-PBF technique in Ti-6Al-4V with similar printing and testing conditions. The reference curve, reported as ASED ($\Delta \bar{W}$) versus number of cycles (N_f_), can be computed from the Whöler fatigue curve using (5):

5 $${\Delta \bar{W}}_{1,{plain}}=\frac{1}{2E}{\Delta \sigma }_{{cr}}^{2}$$

where E is the Young’s Modulus, and $\Delta {\sigma }_{{cr}}$ is the full range plain fatigue limit at a given load ratio R. Once defined the reference curve, the obtained ASED values at the 95% quantile can then quantitatively inform the curve, checking whereas the predicted ASED falls or not within the curve scatter bands. The latter were calculated with a ± 1.5 factor, which in accordance with^45^ corresponds to an approximately 22% error band on the stress amplitude.

Fatigue resistance can be rooted to the interplay of two factors: the material resistance and the load severity. These factors are locally combined, and the result may lead to fatigue failure or component safety. The interplay is hard to quantify in a local computationally efficient manner and therefore, a statistical approach can be applied. The material resistance is commonly presented as a random variable characterised by the specimens’ dispersion during fatigue tests. In this analysis, the load severity can also be presented as a random variable, with mean equal to ${\Delta \bar{W}}_{95 \% }$ and with confidence intervals as per (4). In this sense, the fatigue life estimation can be posed in terms of reliability. This parameter is computed thanks to a Monte Carlo approach as follows: two populations composed by 50000 individuals, one representing the material resistance and one the load severity, are generated. These populations leverage on the hypothesis of a Gaussian distribution of the material resistance and of the load severity, characterized by the statistic descriptors obtained respectively experimentally and numerically. The individuals ${\Delta \bar{W}}_{S}$ belonging to the material resistance population are randomly pared with one ${\Delta \bar{W}}_{\sigma }$ individual each belonging to the load severity and the two are subtracted computing the margin of safety $m$, as per (6):

6 $$m=\,{\Delta \bar{W}}_{S}-\,{\Delta \bar{W}}_{\sigma }$$

The margin of safety is itself a novel population following the Gaussian distribution. The reliability is computed as the complement to 1 of the cumulative probability associated with 0 of this distribution^46^.

### Experimental fatigue tests

Fatigue tests on Ti-6Al-4V thin-strut specimens were performed using a Rumul Mikroton fatigue machine equipped with a 1 kN load cell. All tests were conducted under load control in a fully reversed loading condition (stress ratio *R* = −1) using a sinusoidal waveform at an operating frequency of 110 Hz. The applied force amplitude was selected based on reference S–N data^19^, with the aim of inducing failure at approximately 1.5 × 10⁵ cycles.

A Zeiss Supra 40 (Zeiss, Germany) field-emission scanning electron microscope (FE-SEM) was employed to examine the fatigue fracture surfaces of the fatigue-failed specimens. Images were acquired in secondary electron mode at an accelerating voltage of 5.00 kV. Prior to analysis, specimens were ultrasonically cleaned in ethanol for 10 min to remove loose debris.

## Supplementary information

Supplementary information

## Data Availability

The datasets generated and/or analysed during the current study are not publicly available due to their integration within ongoing related studies, but are available from the corresponding author on reasonable request. The underlying code for this study is not publicly available but may be made available to qualified researchers on reasonable request from the corresponding authors.

## Code availability

The underlying code for this study is not publicly available but may be made available to qualified researchers on reasonable request from the corresponding authors.
