# Supplementary material for: Statistical average strain energy density fatigue estimation of strut-based metamaterials via synthetic as-built CAD digital twins
Source: NPJ Metamater. 2026 Jun 2;2(1):19. doi: 10.1038/s44455-026-00030-z (PMC13229937; doi:10.1038/s44455-026-00030-z)
Supplement: Supplementary file 1 — Supplementary information [file 44455_2026_30_MOESM1_ESM.pdf]

Supplementary Information A

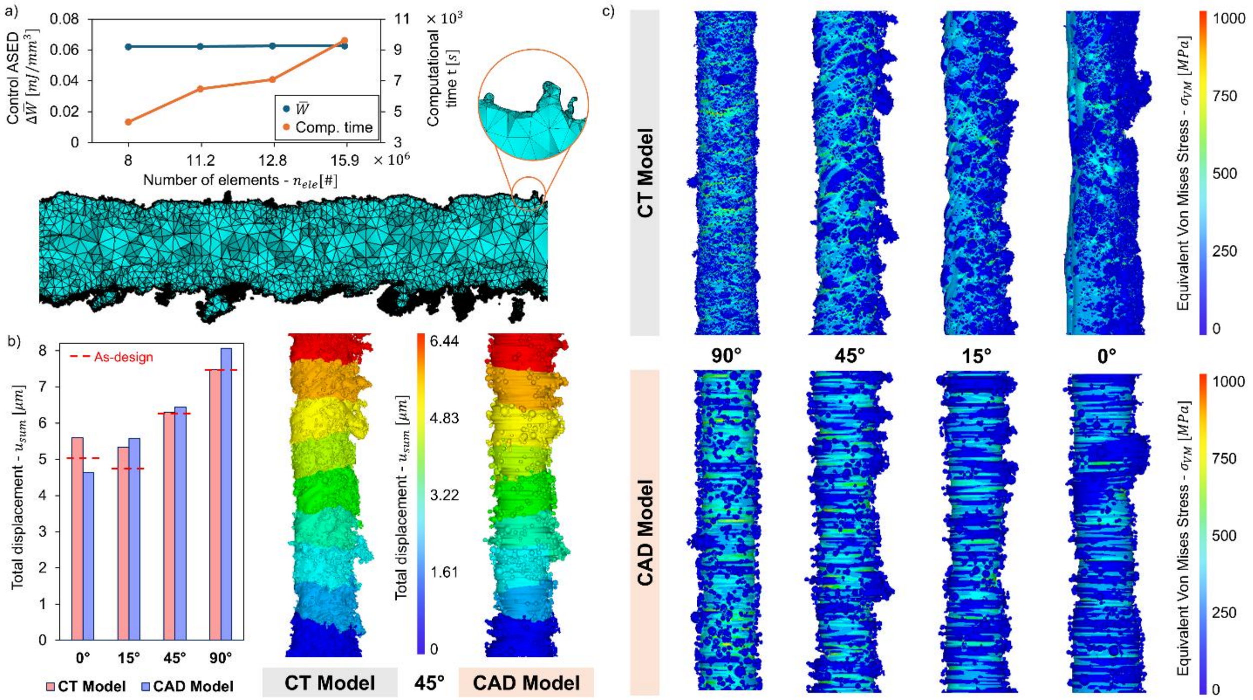

Figure S1: a) Mesh convergence example for the finite element simulation of thin strut reconstructed geometries. b) Comparison of the displacement fields and the maximum displacements between micro-CT reconstructed geometries and synthetic CAD models. c) Direct comparison between the equivalent Von Mises stress distribution for micro-CT reconstructed geometries and synthetic CAD models.

Figure S1a depicts the mesh convergence study and the resulting finite element mesh obtained for the CT-based geometry reconstructions and for the synthetic as-built CAD geometries. In both cases, the meshing parameters are provided after a convergence case study, where displacements, computational time and a control point for Average Strain Energy Density (ASED) calculation are considered. The reconstructed geometry of the 45° specimen is selected as representative case study for the entire simulation set and different finite element models characterized by variable mesh parameters are conducted. The meshing process is conducted using the nTop v5.36.2 (nTopology, USA) software. The mesh is highly refined on the specimen surface to improve the precision on the local defects, and its dimension is kept constant with the same refinement degree over a given thickness to ensure stability in the ASED calculation. After this initial step, the mesh dimension is gradually increased moving radially toward the specimen axis to reduce the computational cost. In the convergence analysis the influence of the parameters for the mesh dimension is studied along with the influence of the decimation on the CT-reconstructed mesh geometry. The most important metric in this convergence analysis is the control ASED: one critical point is arbitrarily selected in the geometry and the ASED computations are performed. Results, displayed in Figure S1a, show consistency in the control ASED calculations even for a reduced number of elements which is directly linked to a more efficient computation. The optimal parameter configuration is the one minimizing the computational time and it is presented in Figure

S2a; the associated mesh parameters are listed in Table S1. Finite element linear elastic simulations are performed in ANSYS Mechanical APDL v2024R1 (ANSYS, USA) with quadratic solid elements SOLID187.

Table S1 - Mesh parameters for optimal simulation performances

**Mesh parameters**	
Decimation	80%
Surface mesh dimension	0.05 mm
Constant mesh thickness	0.1 mm
Inner mesh inflation rate	20

Figure S1b depicts the deformations associated with each CT-synthetic CAD couple. It can be noticed a substantial agreement between the two models, with the synthetic CAD more compliant with respect to the CT geometrical reconstruction in 15°, 45°, and 90° configurations. This behaviour is linked to the different representation of the parasitic masses between the synthetic CAD and the CT geometry: this contribution is lightly underestimated in the CAD reconstruction. An opposite trend is registered instead for the 0° configuration, where the parasitic masses identified on the lowerskin of the strut are assimilated to a bulk cross-section by the synthetic CAD, generating a stiffer model with respect to the CT-based one. The effect of the parasitic masses in the as-built geometries can be also observed comparing the displacement results of both as-built models with the ones obtained in the as-designed configuration. The 45° and 90° configurations are characterized by a larger adherence between the as designed and the CT simulations, and this can be rooted to the more favourable printing orientation of these struts [1]. The 15° and 0°, in contrast, show a larger deviation, symptomatic of a larger geometrical discrepancy between the as-built and as-designed geometries.

Figure S1c depicts the equivalent Von Mises stress distribution of the CT reconstructed models and the synthetic CAD ones. While a global coherence is found among the different models, specific patterns are visible. In the synthetic CAD models, the ridges characteristic of the generation, act as stress raiser giving to the specimen a peculiar stress pattern orthogonal to the specimen axis. CT-based geometries instead are characterized by a stress distribution deriving from the specimen waviness, with specific inclination rooted in the specimen manufacturing process.

Supplementary Information B

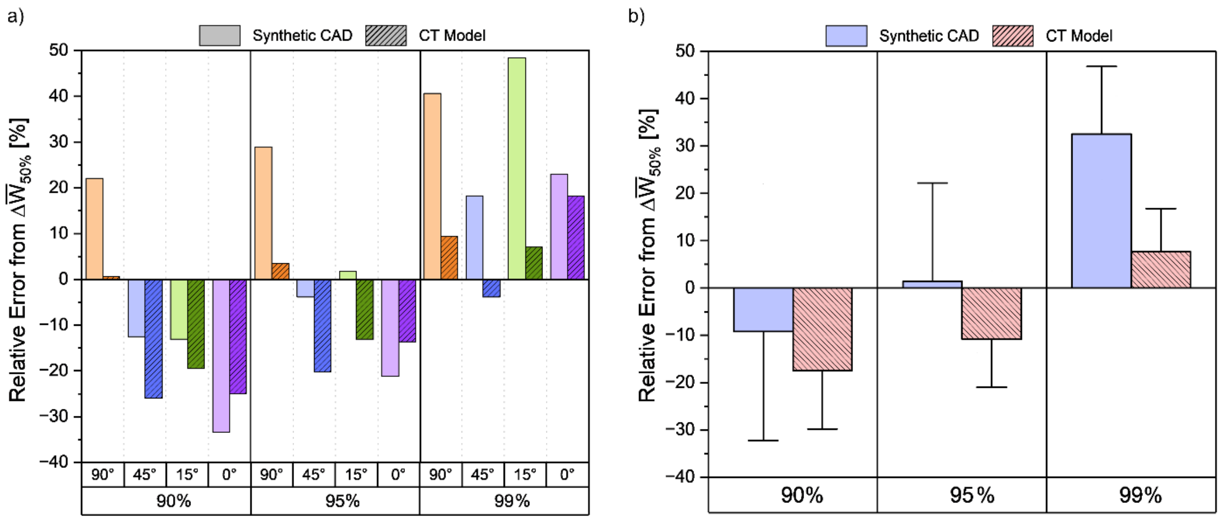

Figure S2 - a) Relative error of the predicted $\Delta\overline{W}$ at probability quantiles 90%, 95%, and 99% with respect to the experimentally determined $\Delta\overline{W}_{50\%}$ from the reference $\Delta\overline{W} - N_f$ curve, broken down by building orientation (0°, 15°, 45°, 90°) and model type (Synthetic CAD vs. CT Model). (b) Mean and scatter of the relative error across all orientations at the 90%, 95%, and 99% quantiles for both model types.

A sensitivity analysis was performed to evaluate the influence of the POT probability quantile on the ASED-based fatigue predictions. Three quantiles were considered: 90%, 95%, and 99%. Figure S2a reports the relative error of the predicted $\Delta\overline{W}$ with respect to the experimentally determined $\Delta\overline{W}_{50\%}$ from the reference $\Delta\overline{W} - N_f$ curve, for each building orientation and model type. Figure S2b aggregates these results across all orientations, showing the mean relative error and its scatter for both the synthetic as-built CAD and CT-based models at each quantile. The 90% quantile yields non-conservative predictions, underestimating the fatigue-critical ASED for the majority of orientations and model types. Conversely, the 99% quantile produces overly conservative estimates, with relative errors exceeding +40% in several cases, which would result unrealistic for capturing the actual fatigue damage phenomenon. The 95% quantile yields relative errors that remain contained and well-distributed around the experimental reference, suggesting it as the most realistic and balanced choice for fatigue modelling. This result is in line with the findings of Raghavendra et al.[2], who employed statistics of extremes applied to SED-based fatigue life predictions for L-PBF lattice metamaterials, demonstrating how the 95% quantile closely aligns with experimentally derived data.

Supplementary Information C

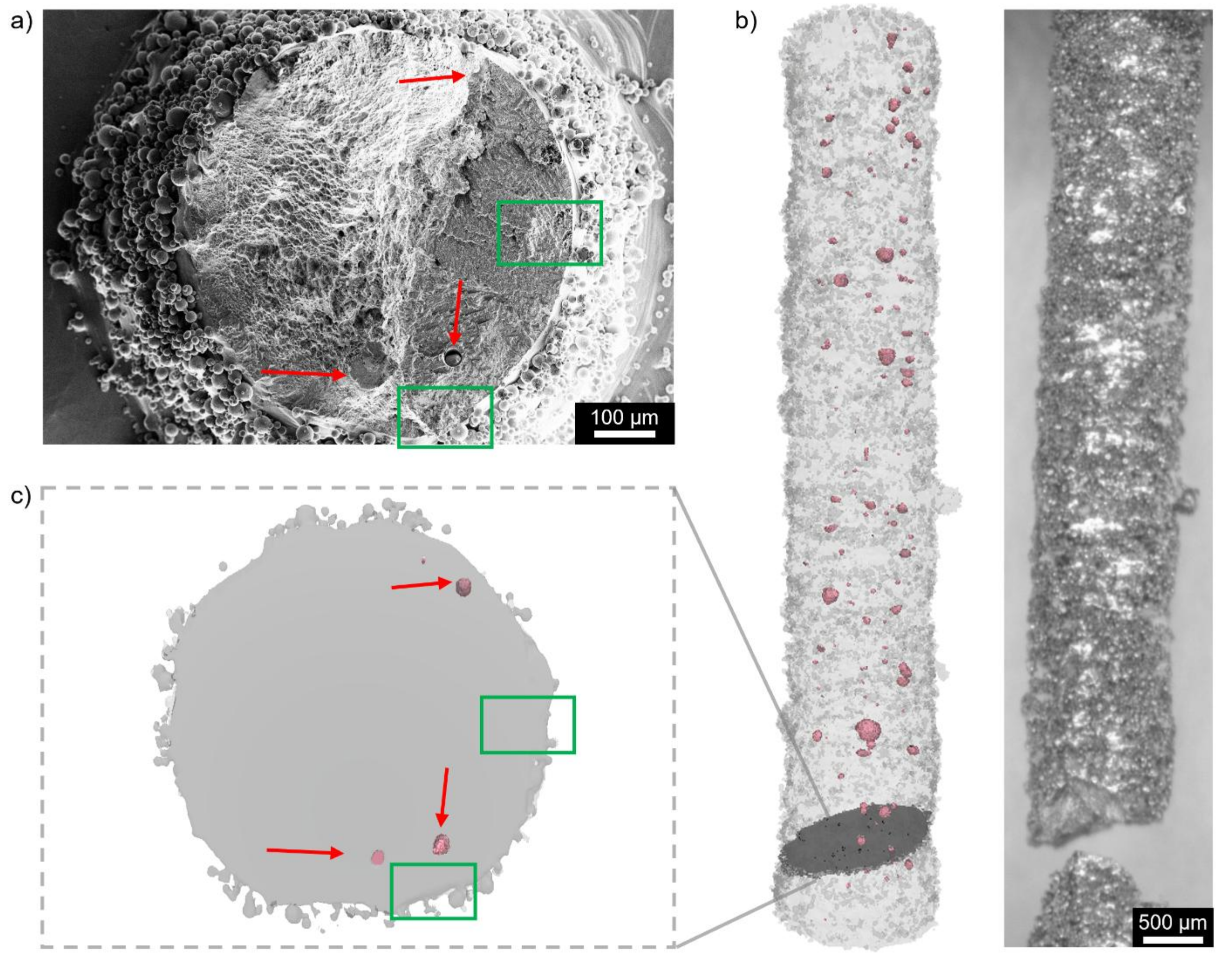

Figure S3 - Characterization of the fracture surface of the 90° strut. (a) SEM fracture surface. Scale bar set at 100 µm. (b) Axial failure location shown by stereomicroscopy (right) and corresponding CT-reconstructed strut (left). The latter shows in pink the CT-detected internal porosity. The scale bar for the stereomicroscopy image is set at 500 µm. (c) CT-derived cross-section extracted at the fracture plane, with internal porosity rendered in pink. Red arrows in (a) and (c) indicate subsurface pores, while green boxes highlight surface micro-notches associated with fatigue crack initiation.

Figure S3 provides a complementary visualization of the fracture site of the 90° struts discussed in *Section Framework's Computational Time*. Figure S3a shows the same SEM fracture surface reported in Figure 4a at a larger field of view, enabling direct comparison with the CT-derived cross-section extracted at the same axial position along the gauge length (Figure S3c). In Figure S3c, internal porosity resolved by CT scanning prior to fatigue testing is shown in pink. Red arrows in Figures S3a and S3c identify subsurface pores exposed at the fracture plane, whereas green boxes highlight surface micro-notches associated with fatigue crack initiation.

Figure S3b locates the failure location within the strut's gauge length. The stereomicroscopy image (right) shows the fracture position on the tested specimen, while the corresponding CT-

reconstructed 90° strut is shown on the left, with the surface rendered transparent to visualize the internal porosity distribution and the fracture zone (darker grey).

References:

1. Murchio, S., Du Plessis, A., Luchin, V., Maniglio, D. & Benedetti, M. Influence of mean stress and building orientation on the fatigue properties of sub-unital thin-strut miniaturized Ti6Al4V specimens additively manufactured via Laser-Powder Bed Fusion. *Int. J. Fatigue* **180**, (2024).
2. Raghavendra, S. *et al.* A probabilistic average strain energy density approach to assess the fatigue strength of additively manufactured cellular lattice materials. *Int. J. Fatigue* **172**, (2023).
